# Association between polymorphisms in mannose-binding lectin 2 gene with pulmonary tuberculosis susceptibility

**DOI:** 10.1186/s41065-020-00146-w

**Published:** 2020-08-03

**Authors:** Wenhao Shen, Li Xiao, Yang Li, Daming Zhou, Wei Zhang

**Affiliations:** 1 Department of Oncology, Taizhou People’s Hospital, Taizhou, 225300 China; 2 Department of Infectious Disease, Taizhou People’s Hospital, Taizhou, 225300 China

**Keywords:** Mannose-binding lectin, Pulmonary tuberculosis, Polymorphism, Meta-analysis, Susceptibility

## Abstract

**Background:**

Mannose-binding lectin (MBL2) is considered to play a role in the human innate immune response to tuberculosis (TB) infections, and 4 common single nucleotide polymorphisms (SNPs) may be associated with pulmonary tuberculosis (PTB) risk. To examine these potential associations, we performed a comprehensive analysis to assess the relationships between *MBL2* polymorphisms and PTB.

**Methods:**

The PubMed, Embase, and SinoMed databases were searched for articles published prior to June 13, 2019. Odds ratios with 95% confidence intervals were calculated to evaluate the strength of the relationships.

**Results:**

There were 37 case-control studies examining the effects of the four SNPs in *MBL2* on PTB. A positive association between rs11003125 and PTB risk was observed in the hospital-based subgroup. Moreover, for the combined polymorphism and PTB risk, positive associations were detected not only in the total population but also in those with Asian origins across all source of control subgroups. No associations were found for rs7096206 or rs7095891.

**Conclusions:**

Our current study indicated that several SNPs in *MBL2* may be associated with susceptibility to PTB.

## Background

Tuberculosis (TB) is a global public health issue that poses serious threats to human health. It has been estimated that 1/3 of the world’s population may be infected with tubercle bacilli, but only 1/10 of individuals infected with *Mycobacterium tuberculosis* go on to develop TB [1], suggesting that there are inherent individual differences in susceptibility to TB that may be related to nutrition, constitution, specific and nonspecific resistance, and genetic susceptibility [2–6]. In fact, many studies have focused on the genetic variations within genes that increase the risk of TB [7, 8]. Previous case-control association studies have revealed that several human genes might be correlated with TB in certain populations. These genes include *interferon-gamma* (*IFNG*), *vitamin D receptor* (*VDR*), *solute carrier family 11a member 1* (*SLC11A1*, which is also known as *NRAMP1*), and *mannose-binding lectin* (*MBL2*) [9–12].

The *MBL2* gene, which is a member of the complement system, has been hypothesized to play a dual role in the innate immune response to infections by activating the classical lectin pathway and by phagocytosis [13, 14]. MBL and other soluble pattern recognition molecules [collectin-10, collectin-11, and ficolins (ficolin-1, ficolin-2, and ficolin-3)] act as mediators of host defense and participate in the maintenance of tissue homeostasis. They can bind to conserved pathogen-specific structures and altered self-antigens, and they form complexes with pentraxins to modulate innate immune functions. All these molecules exhibit distinct expressions in different tissue compartments, but all of them are found to varying degrees in the circulatory system. A common feature of these molecules is their ability to interact with a set of serine proteases named MASPs (MASP-1, MASP-2, and MASP-3) [15]. Human MBL is encoded by *MBL2* on chromosome 10 (10q11.2-q21; OMIM 154545), which comprises four exons. *MBL2* is reported to have several genetic polymorphisms that are commonly associated with MBL serum levels. Three point substitutions, located at codons 52, 54, and 57 in exon 1, are supposed to disrupt the assembly of MBL trimers or accelerate the degradation of the protein, thereby causing a decrease in the functional activity of MBL in the serum. These mutations are frequently referred to as variants D, B, and C, respectively, and they are collectively known as O, while A is the wild type. In addition, three other point substitutions have been reported in the nonstructural region: two at positions − 550 (H/L variants) and − 221 (X/Y variants) in the promoter region and one point mutation at position − 4 (P/Q variants) in the 5′-untranslated (UTR) region [16–18].

Many epidemiologic studies, including meta-analyses, suggest that there are relationships between *MBL2* gene variations and pulmonary TB (PTB) risk [10, 16, 17, 19–47]. However, ambiguous conclusions have been reported; thus, it is necessary to perform an undated meta-analysis that includes a reanalysis of relevant studies.

## Materials and methods

### Search strategy and criteria

The PubMed, Embase, and SinoMed databases were searched for articles published prior to June 13, 2019, using the keywords “tuberculosis,” “TB,” “polymorphism,” and “mannose binding lectin 2 or *MBL2*”. A total of 163 papers were identified, 30 of which were consistent with our criteria. The inclusion criteria for papers were as follows: (i) examined the relationship between PTB susceptibility and *MBL* variations, (ii) case-control study, and (iii) contained a complete number of genotypes (MM + MW + WW) among cases and controls. The exclusion criteria were as follows: (i) no control group, (ii) incomplete genotype frequency data, (iii) duplicate publication, and (iv) controls did not meet the Hardy-Weinberg equilibrium (HWE) standards.

### Data extraction

The essential data are listed as follows: first author name, publication year, original country, race, total samples of case/control, each genotype in cases/controls, source of control and genotype methods. Race was classified as Caucasian, Asian, African, or mixed. The source of control subgroups included population-based (PB) and hospital-based (HB) subgroups. The type of TB included total TB, PTB, and EPTB.

### Quality score assessment (NOS)

The NOS was used to assess the quality of each study and to assess the various aspects of the methodology, including the selection of cases, the comparability of groups and the determination of exposure. The total score on the NOS ranges from 0 to 9 stars. Studies with scores greater than 7 are considered high-quality studies [48].

### Statistical analysis

We used 95% CIs to measure the correlation between SNPs in *MBL2* and PTB risk based on the genotype frequency of the case and control groups. The *Z*-test was used to determine the statistical significance of the correlations. The heterogeneity between the studies was evaluated using a *Q*-test based on the χ^2^ method. In the *Q*-test, a *P* value greater than 0.05 indicates that there is a lack of heterogeneity between the studies. Because the *Q*-statistic does not reveal the statistical significance of the heterogeneity, the *I*^2^ test was applied to better assess the extent of heterogeneity. As a guide, *I*^2^ values are divided into three categories (≤25%, 25–50%, ≥50%), corresponding to low risk, medium risk, and high risk, respectively [49]. If *P* ≤ 0.05 or *I*^2^ ≥ 50%, a random effects model was adopted; otherwise, a fixed effects model was used [50, 51]. We accessed the association between SNPs in *MBL2* and PTB risk by testing the allelic contrast (X versus Y for rs7096206; L versus H for rs11003125; Q versus P for rs7095891; and O versus A for A/O combined SNP), heterozygote comparison (XY versus YY for rs7096206; LH versus HH for rs11003125; QP versus PP for rs7095891; and OA versus AA for A/O combined SNP), homozygote comparison (XX versus YY for rs7096206; LL versus HH for rs11003125; QQ versus PP for rs7095891; and OO versus AA for A/O combined SNP), recessive genetic model (XX versus XY + YY for rs7096206; LL versus LH + HH for rs11003125; and QQ versus QP + PP for rs7095891; OO versus OA + AA for A/O combined SNP) and dominant genetic model (XX + XY versus YY for rs7096206; LL + LH versus HH for rs11003125; QQ + QP versus PP for rs7095891; and OO + OA versus AA for A/O combined SNP). Sensitivity analysis was applied to assess the stability of the results. The HWE was evaluated by Pearson’s χ^2^ test, and *P* = 0.05 was considered statistically significant [52]. Publication bias was assessed by both Egger’s and Begg’s tests [53]. All statistical tests were carried out by version 11.0 of the Stata Software (StataCorp LP, College Station, TX, USA).

### Genotyping methods

Methods for genotyping the SNPs in *MBL2* were derived from the literature in Table 1.

**Table 1:** Basic information of the association between 4 SNPs in *MBL2* and TB, especially for PTB susceptibility

First author	Year	Origin	Ethnicity	Source of	Type	Case	Control	Case			Control				Method	NOS
**Ref No**				**Control**				**MM**	**MW**	**WW**	**MM**	**MW**	**WW**			
**rs7096206**								XX/GG	XY/GC	YY/CC	XX/GG	XY/GC	YY/CC	HWE		
Liu [10]	2006	China	Asian	PB	PTB	141	212	6	44	91	7	54	151	0.43	PCR-SSP/PCR-SSOP	7
Wu	2017	China	Asian	HB	PTB	151	453	7	47	97	15	120	318	0.379	PCR-RFLP/PCR-SSCP	6
Thye [41]	2011	Germany	Caucasian	PB	PTB	1859	2180	26	396	1437	31	486	1663	0.503	DASH-FRET	7
Feng [30]	2016	China	Asian	HB	PTB	99	89	0	9	90	1	26	62	0.336	Taqman	5
Liu	2015	China	Asian	HB	PTB	112	120	11	35	66	2	40	78	0.215	PCR-RFLP	7
Chen [24]	2014	China	Asian	PB	PTB	205	216	5	77	123	8	49	159	0.098	PCR-SSP	7
Wang [42]	2009	China	Asian	PB	PTB	449	249	12	122	315	7	61	181	0.503	AMLR	7
Alagarasu [19]	2007	India	Asian	HB	total	109	146	3	44	62	13	61	72	0.987	PCR-SSP	6
Chen [25]	2015	China	Asian	PB	total	503	419	12	166	325	10	113	296	0.839	PCR-SSP	8
Amiri [20]	2017	Iran	Asian	PB	PTB	100	100	5	36	59	7	29	64	0.159	PCR-SSP	7
Cruz [27]	2013	Brazil	Caucasian	HB	PTB	119	148	4	40	75	6	32	110	0.076	Sequencing	6
Cruz [27]	2013	Brazil	Caucasian	HB	EPTB	36	148	1	9	26	6	32	110	0.076	Sequencing	6
**rs11003125**								LL	LH	HH	LL	LH	HH	HWE		
Liu [10]	2006	China	Asian	PB	PTB	141	212	31	66	44	58	105	49	0.911	PCR-SSP/PCR-SSOP	7
Thye [41]	2011	Germany	Caucasian	PB	PTB	1843	2174	7	265	1571	9	287	1878	0.577	DASH-FRET	7
Feng [30]	2016	China	Asian	HB	PTB	99	89	28	45	26	21	36	32	0.092	Taqman	5
Li [34]	2011	China	Asian	PB	PTB	231	226	34	92	105	31	106	89	0.949	PCR-SSP	7
Wu	2017	China	Asian	HB	PTB	151	453	41	64	46	104	248	101	0.043	PCR-RFLP/PCR-SSCP	6
Zhou [46]	2011	China	Asian	HB	PTB	226	141	58	101	67	42	10	89	< 0.01	PCR-SSP	6
Zhang [44]	2011	China	Asian	HB	PTB	220	213	29	75	116	51	76	86	< 0.01	PCR-SSP	6
Wang [42]	2009	China	Asian	PB	PTB	449	249	91	235	123	60	108	81	0.046	AMLR	7
Amiri [20]	2017	Iran	Asian	PB	PTB	100	100	22	43	35	30	48	22	0.735	PCR-SSP	7
Cruz [27]	2013	Brazil	Caucasian	HB	PTB	119	148	66	45	8	68	61	19	0.367	Sequencing	6
Cruz [27]	2013	Brazil	Caucasian	HB	EPTB	36	148	16	18	2	68	61	19	0.367	Sequencing	6
**rs7095891**								QQ	QP	PP	QQ	QP	PP	HWE		
Liu [10]	2006	China	Asian	PB	PTB	141	212	1	22	118	2	39	171	0.891	PCR-SSP/PCR-SSOP	7
Wu	2017	China	Asian	HB	PTB	151	453	1	26	124	2	87	364	0.181	PCR-RFLP/PCR-SSCP	6
Thye [41]	2011	Germany	Caucasian	PB	PTB	1953	2230	308	920	725	319	1086	825	0.205	DASH-FRET	7
Zhou [46]	2011	China	Asian	HB	PTB	226	231	24	90	112	25	89	117	0.201	PCR-SSP	6
Feng [30]	2016	China	Asian	HB	PTB	99	89	7	34	58	0	24	65	0.141	Taqman	5
Wang [42]	2009	China	Asian	PB	PTB	449	249	3	114	332	2	64	183	0.155	AMLR	7
Zhang [44]	2011	China	Asian	HB	PTB	220	213	17	31	172	21	36	156	< 0.01	PCR-SSP	6
Amiri [20]	2017	Iran	Asian	PB	PTB	100	100	3	21	76	5	26	69	0.233	PCR-SSP	7
**AA/AO/OO**								OO	OA	AA	OO	OA	AA	HWE		
Garcia-Laorden [17]	2006	Spain	Caucasian	HB	total	106	344	3	33	70	27	134	183	0.721	PCR-RFLP	6
Søborg	2003	Denmark	Caucasian	PB	total/ White	59	250	4	18	37	7	86	157	0.235	PCR-SSP	8
Søborg	2003	Denmark	Caucasian	PB	total/ Nonwhite	50	250	4	12	34	7	86	157	0.235	PCR-SSP	8
Capparelli [22]	2009	Italy	Caucasian	HB	PTB	274	288	61	158	55	10	112	166	0.087	Sequencing	6
Garcıa-Gasalla [32]	2014	Spain	Caucasian	HB	total	76	106	4	24	48	1	34	71	0.156	PCR-SSP	6
Alagarasu [19]	2007	India	Asian	HB	total	275	146	25	87	145	7	53	86	0.747	PCR-SSP	6
Zhao [45]	2014	China	Asian	PB	PTB	900	870	101	279	520	53	303	514	0.352	PCR-RFLP	7
Li [34]	2011	China	Asian	PB	PTB	231	226	3	57	171	3	37	186	0.461	PCR-SSP	7
Li [33]	2009	China	Asian	HB	PTB	141	152	6	56	79	8	38	106	0.075	PCR-SSP	6
Liu [10]	2006	China	Asian	PB	PTB	141	212	4	34	103	4	42	166	0.487	PCR-SSP/PCR-SSOP	7
Zhou [47]	2012	China	Asian	HB	PTB	226	231	14	106	106	5	80	146	0.114	PCR-SSP	6
Liu	2015	China	Asian	HB	PTB	112	120	3	29	80	2	22	96	0.576	PCR-RFLP	7
Fang [29]	2011	China	Asian	HB	PTB	100	100	1	25	74	0	25	75	0.153	PCR-RFLP	6
Wu	2017	China	Asian	HB	PTB	151	454	2	37	112	8	97	348	0.681	PCR-RFLP/PCR-SSCP	6
Singla	2011	India	Asian	HB	PTB	286	397	11	100	175	35	155	207	0.441	PCR-RFLP	6
Singla	2011	India	Asian	HB	EPTB	71	397	2	26	43	35	155	207	0.441	PCR-RFLP	6
Özbaþ-Gerçeker	2003	Turkey	Caucasian	PB	PTB	49	100	0	9	40	4	20	76	0.09	PCR	7
Wit [28]	2011	South Africa	African	PB	total	499	313	2	134	363	0	102	211	< 0.01	PCR-RFLP	8
Feng [30]	2016	China	Asian	HB	PTB	381	267	14	177	190	12	176	79	< 0.01	Taqman	5
Wang [42]	2009	China	Asian	PB	PTB	449	249	4	133	312	3	82	164	0.038	AMLR	7
Thye [41]	2011	Germany	Caucasian	PB	PTB	1893	1040	193	815	885	126	426	488	0.029	DASH-FRET	7
Ceylan [23]	2017	Turkey	Caucasian	HB	total	69	70	8	13	48	12	11	47	< 0.01	PCR-RFLP	7
Amiri [20]	2017	Iran	Asian	PB	PTB	100	100	2	29	69	1	27	72	0.374	PCR-SSP	7
Cruz [27]	2013	Brazil	Caucasian	HB	PTB	119	148	7	41	71	6	34	108	0.129	Sequencing	6
Cruz [27]	2013	Brazil	Caucasian	HB	EPTB	36	148	1	14	21	6	34	108	0.129	Sequencing	6
Selvaraj [37]	1999	India	Asian	PB	PTB	202	109	22	73	107	2	39	68	0.175	PCR-RFLP	7
Araújo	2013	Brazil	Caucasian	HB	PTB	133	159	2	47	84	2	56	101	0.058	PCR	6
Araújo	2013	Brazil	Caucasian	HB	EPTB	34	159	1	15	18	2	56	101	0.058	PCR	6
Fitness [31]	2004	UK	Caucasian	PB	total	322	546	12	105	205	24	160	362	0.245	fluorescence PCR/ARMS-PCR	7
Søborg	2007	Denmark	Caucasian	PB	PTB	443	432	22	132	289	30	131	271	0.013	PCR-RFLP/PCR-SSP	7

*HWE* Hardy-Weinberg equilibrium; *M* Mutated allele; *W* Wide type allele; *HB* Hospital-based; *PB* Population-based; *TB* Tuberculosis; *PTB*: Pulmonary TB, *EPTB* Extra-pulmonary TB; *PCR-FLIP* Polymerase chain reaction and restrictive fragment length polymorphism; *SSP* Sequence specific primer; *SSOP* Sequence-specific oligonucleotide probe; *SSCP* Single-strand conformation polymorphism; *DASH-FRET* Dynamic allele-specific hybridization with fluorescence resonance energy transfer; *AMLR* Allelic-specific multiplex ligase-detection reaction; *ARMS* Amplification refractory mutation system; *NOS* Newcastle-Ottawa scale

## Results

### Study characteristics

**Fig. 1:**
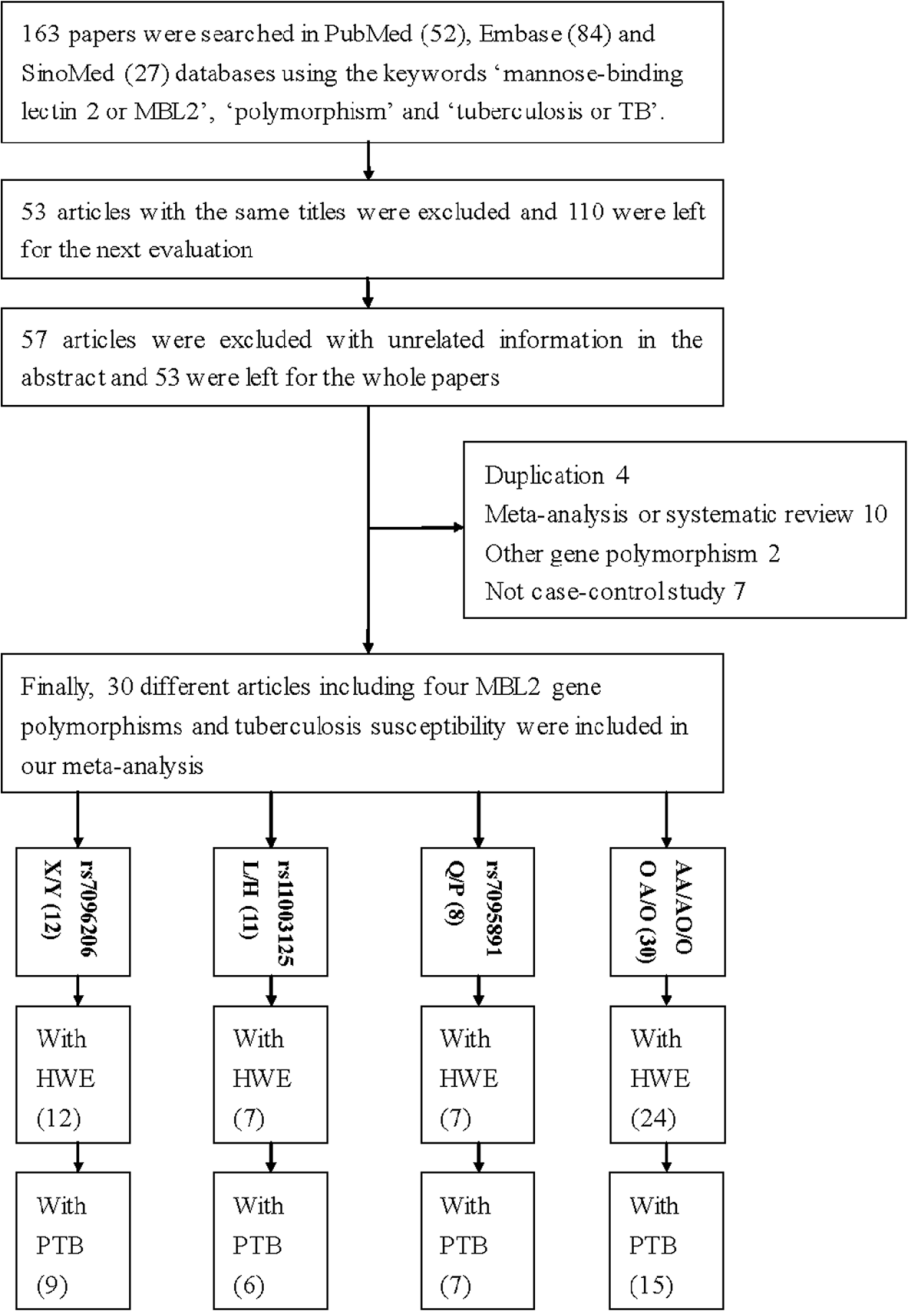
A flowchart illustrating the search strategy for identifying related studies. HWE: Hardy-Weinberg equilibrium; PTB: pulmonary tuberculosis

A total of 163 articles were retrieved from the PubMed, Embase, and SinoMed databases by using various combinations of the abovementioned keywords. Fifty-three duplicate articles were removed after screening the titles, as shown in Fig. 1. Another 57 articles were removed because they did not contain relevant information. Next, the full texts of 53 articles were evaluated, and 23 additional articles were excluded because they contained duplicate data (4), they were meta-analyses/systematic reviews (10), they examined polymorphisms in other genes (2), or they were not case-control studies (7). Finally, 30 articles examining the association between the 4 SNPs in *MBL2* and TB susceptibility were included (12 articles for rs7096206, 11 for rs11003125, 8 for rs7095891 and 30 for the A/O SNP). After filtering out studies that met our exclusion criteria, 9 different case-control studies were included for rs7096206, 6 for rs11003125, 7 for rs7095891, and 15 for the A/O SNP (Table 1). Overall, 37 case-control studies with 12,052 cases of PTB as well as 13,905 controls were included [10, 17, 19–47]. The controls were mainly healthy individuals.

### Quantitative synthesis

**Fig. 2:**
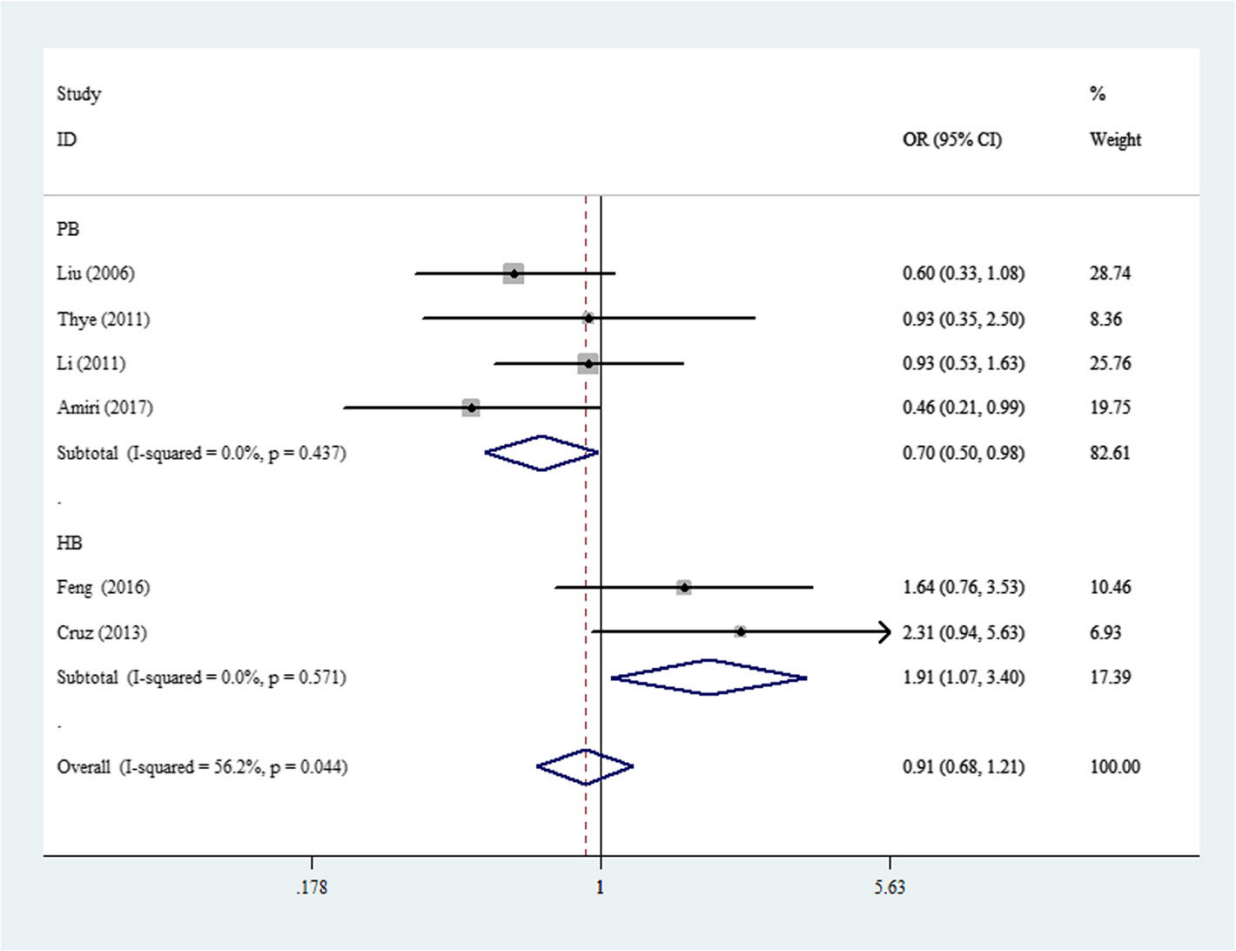
Forest plot of PTB risk associated with *MBL2* rs11003125 polymorphism (LL vs. HH) in the subgroup about source of control. Square and horizontal lines correspond to specific OR or 95% CI. The area of the squares reflects the weight (inverse proportional variance). Diamonds represent the total OR or 95% CI

**Fig. 3:**
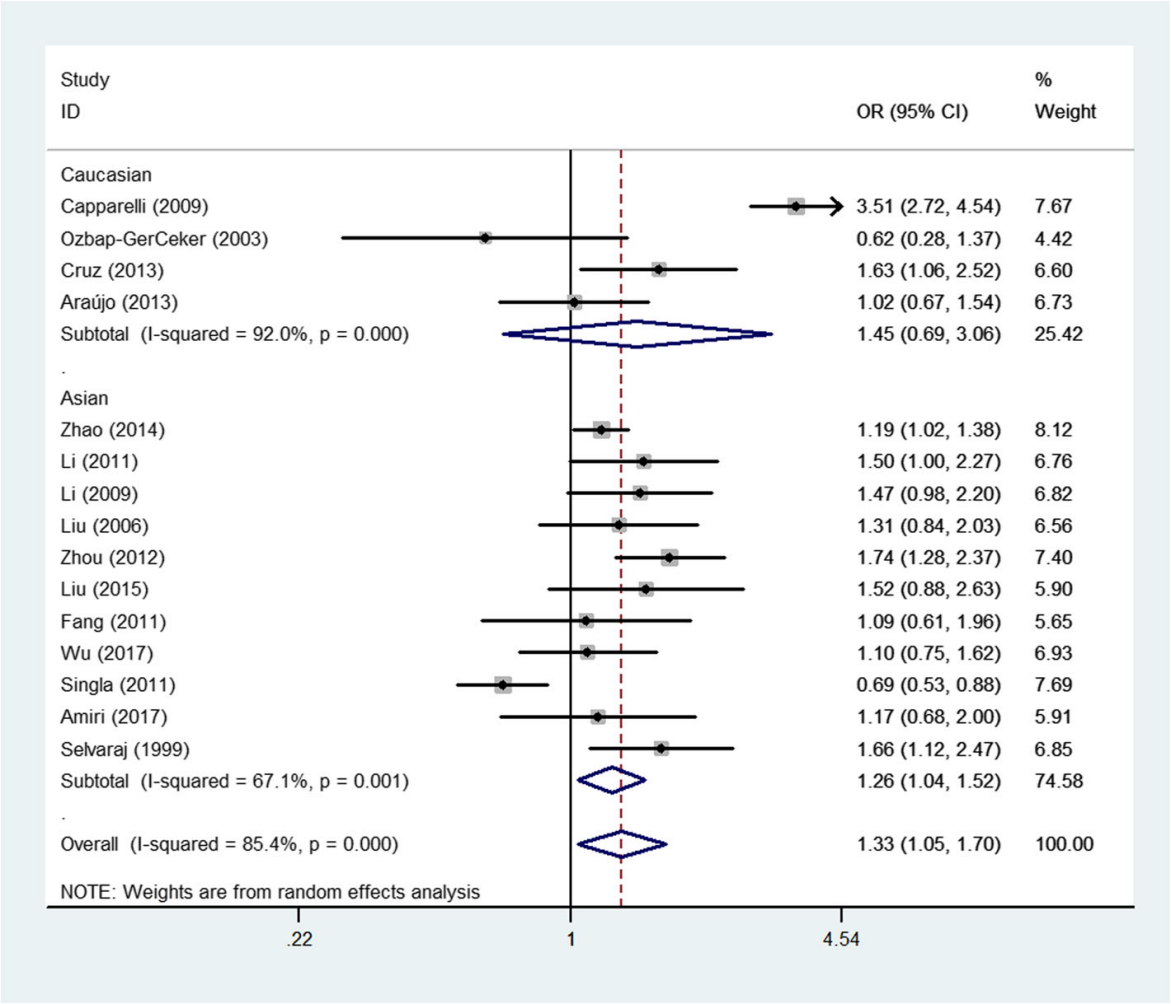
Forest plot of PTB risk associated with *MBL2* A/O combined polymorphism (allelic contrast) by the whole samples and ethnicity

**Fig. 4:**
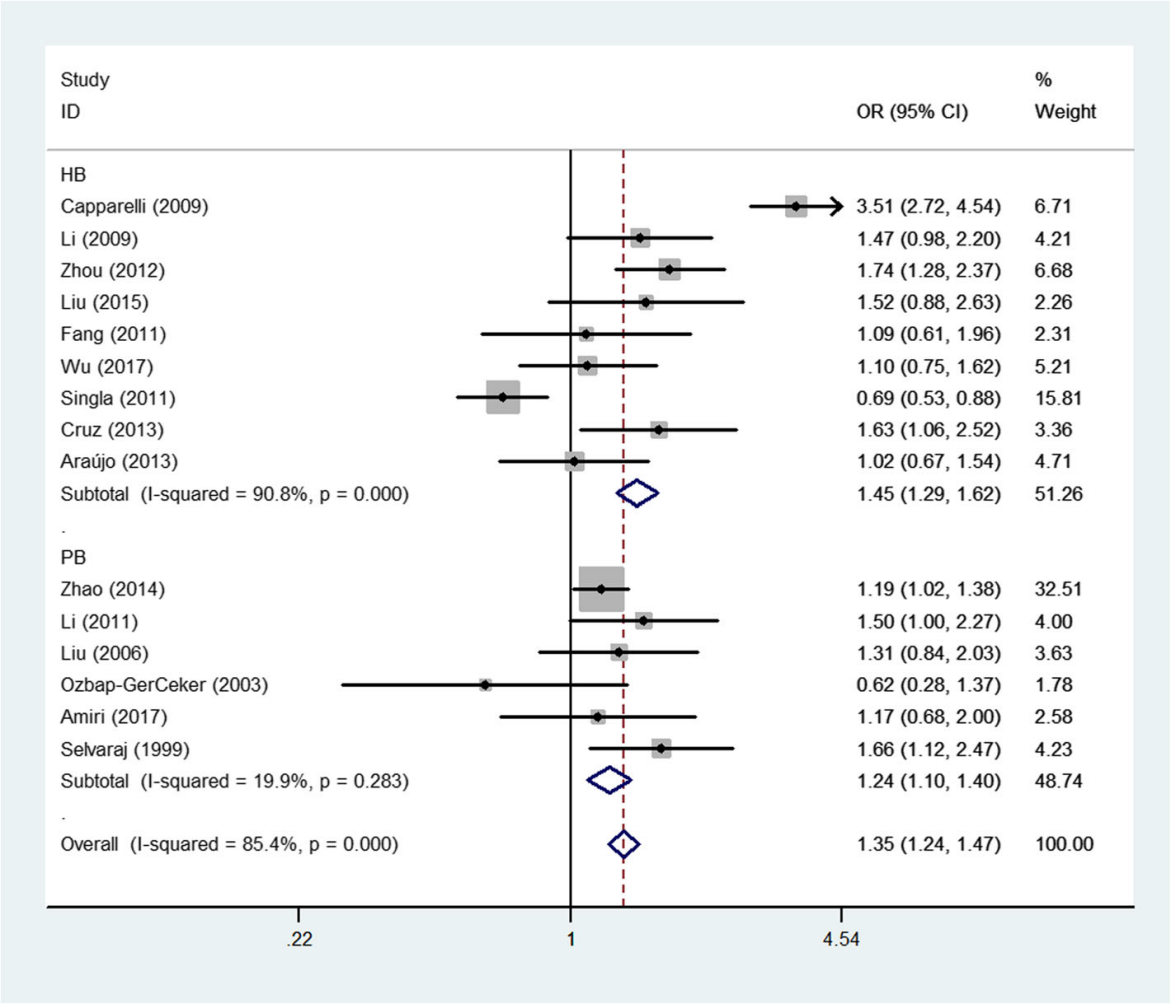
Forest plot of PTB risk associated with *MBL2* A/O combined polymorphism (allelic contrast) by source of control

**Fig. 5:**
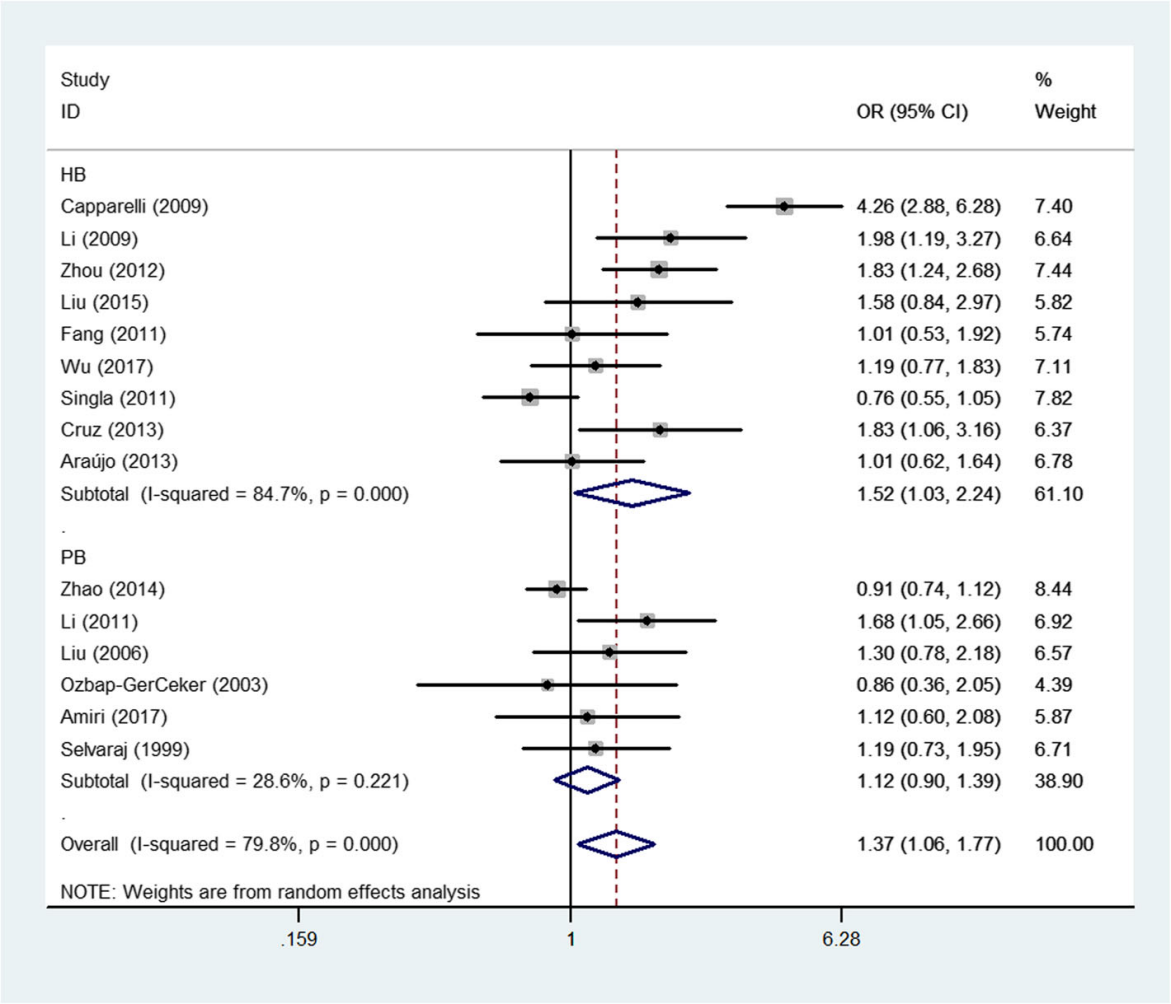
Forest plot of PTB risk associated with *MBL2* A/O combined polymorphism (heterozygote comparison) by source of control

The associations between the 4 SNPs in *MBL2* and PTB risk are shown in Table 2 and Figs. 2, 3, 4, and 5. For rs11003125, although negative associations were found in the total sample and in ethnic subgroups, a positive association was detected in the HB analysis (OR: 1.40, 95% CI: 1.06–1.85, *P*(heterogeneity): 0.755, *P*: 0.017, in the allelic contrast; OR: 1.91, 95% CI: 1.07–3.40, *P*(heterogeneity): 0.571, *P*: 0.029, in the homozygous comparison model (Fig. 2); and OR: 1.73, 95% CI: 1.05–2.86, *P*(heterogeneity): 0.633, *P*: 0.033 in the dominant genetic model).

**Table 2:** Total and stratified subgroup analysis for 4 SNPs in *MBL2* and PTB susceptibility

Variables	N	Case/	OR(95%CI) *P*_h_*P* I^2^(%)	OR(95%CI) *P*_h_*P* I^2^(%)	OR(95%CI) *P*_h_*P* I^2^(%)	OR(95%CI) *P*_h_*P* I^2^(%)	OR(95%CI) *P*_h_*P* I^2^(%)
Author/SNP		Control	M-allele vs. W-allele	MW vs. WW	MM vs. WW	MM + MW vs. WW	MM vs. MW + WW
**rs7096206(XX/XY/YY)**							
Total	9	3235/3767	1.14(0.92–1.40)0.001 0.226 69.0	1.17(0.89–1.53)0.000 0.264 73.5	1.14(0.83–1.57)0.470 0.419 0.0	1.17(0.90–1.51)0.000 0.234 72.5	1.09(0.79–1.50)0.437 0.598 0.0
Ethnicity							
Asian	7	1257/1439	1.13(0.86–1.49)0.005 0.366 68.0	1.13(0.80–1.61)0.002 0.482 71.8	1.30(0.84–1.99)0.308 0.238 16.0	1.15(0.83–1.60)0.002 0.412 70.7	1.20(0.79–1.84)0.264 0.395 21.7
Caucasian	2	1978/2328	1.11(0.75–1.64)0.081 0.608 67.1	1.24(0.65–2.37)0.022 0.504 80.9	0.97(0.60–1.58)0.922 0.908 0.0	1.20(0.68–2.11)0.035 0.531 77.6	0.96(0.59–1.56)0.804 0.864 0.0
Source of control							
HB	4	481/810	1.01(0.58–1.76)0.001 0.973 82.4	0.93(0.48–1.83)0.001 0.840 82.5	1.77(0.96–3.27)0.159 0.069 42.0	0.97(0.51–1.87)0.001 0.935 82.9	1.67(0.91–3.06)0.147 0.099 44.1
PB	5	2754/2957	1.04(0.94–1.16)0.114 0.431 46.2	1.27(0.95–1.70)0.012 0.112 69.1	0.97(0.67–1.42)0.954 0.885 0.0	1.22(0.94–1.59)0.026 0.130 63.9	0.93(0.64–1.35)0.909 0.705 0.0
**rs11003125(LL/LH/HH)**							
Total	6	2533/2949	0.99(0.80–1.22)0.011 0.900 66.2	0.93(0.70–1.25)0.049 0.649 55.1	0.95(0.60–1.49)0.044 0.808 56.2	0.96(0.71–1.29)0.013 0.716 65.3	1.01(0.80–1.28)0.309 0.930 16.3
Ethnicity							
Asian	4	571/627	0.87(0.67–1.13)0.072 0.293 57.1	0.78(0.60–1.01)0.171 0.061 40.1	0.80(0.49–1.31)0.087 0.370 54.4	0.80(0.54–1.17)0.075 0.249 56.5	0.90(0.68–1.19)0.387 0.455 1.0
Caucasian	2	1962/2322	1.14(0.98–1.33)0.162 0.093 48.8	1.12(0.94–1.34)0.330 0.193 0.0	1.55(0.81–2.96)0.182 0.182 43.9	1.13(0.95–1.34)0.168 0.171 47.4	1.34(0.87–2.06)0.405 0.189 0.0
Source of control							
HB	2	218/237	1.40(1.06–1.85)0.755 0.017 0.0	1.61(0.94–2.78)0.822 0.084 0.0	1.91(1.07–3.40)0.571 0.029 0.0	1.73(1.05–2.86)0.633 0.033 0.0	1.40(0.94–2.06)0.741 0.094 0.0
PB	4	2315/2712	0.87(0.70–1.08)0.047 0.217 62.6	0.82(0.59–1.13)0.048 0.219 62.1	0.70(0.50–0.98)0.437 0.039 0.0	0.80(0.57–1.11)0.026 0.179 67.7	0.84(0.62–1.13)0.634 0.204 0.0
**rs7095891(QQ/QP/PP)**							
Total	7	3119/3524	1.02(0.95–1.11)0.166 0.552 34.3	0.97(0.87–1.08)0.713 0.589 0.0	1.10(0.93–1.31)0.586 0.262 0.0	0.99(0.90–1.10)0.389 0.912 4.9	1.12(0.96–1.32)0.641 0.161 0.0
Ethnicity							
Asian	6	1166/1334	1.01(0.86–1.18)0.106 0.924 44.9	0.98(0.81–1.19)0.592 0.843 0.0	1.14(0.70–1.85)0.442 0.589 0.0	0.99(0.82–1.25)0.277 0.942 20.8	1.12(0.70–1.79)0.514 0.648 0.0
Source of control							
HB	3	476/773	1.19(0.79–1.81)0.048 0.406 67.0	1.07(0.82–1.41)0.341 0.608 7.1	1.35(0.77–2.35)0.148 0.290 47.7	1.11(0.86–1.44)0.140 0.423 49.2	1.28(0.75–2.19)0.170 0.367 43.5
PB	4	2643/2791	1.01(0.93–1.10)0.495 0.808 0.0	0.95(0.84–1.07)0.817 0.404 0.0	1.08(0.90–1.30)0.787 0.406 0.0	0.97(0.87–1.09)0.670 0.638 0.0	1.11(0.94–1.31)0.815 0.236 0.0
**AA/AO/OO**							
Total	15	3165/3665	1.33(1.05–1.70)0.000 0.020 85.4	1.37(1.06–1.77)0.000 0.018 79.8	1.82(0.94–3.51)0.000 0.073 79.2	1.41(1.06–1.86)0.000 0.017 84.3	1.61(0.94–2.77)0.000 0.083 69.2
Ethnicity							
Asian	11	2590/2970	1.26(1.04–1.52)0.001 0.017 67.1	1.24(1.01–1.52)0.005 0.044 60.3	1.51(0.85–2.65)0.005 0.157 60.7	1.28(1.04–1.57)0.003 0.021 62.9	1.41(0.82–2.41)0.009 0.216 57.5
Caucasian	4	575/695	1.45(0.69–3.06)0.000 0.331 92.0	1.67(0.76–3.67)0.000 0.197 88.4	2.26(0.35–14.56)0.000 0.393 85.6	1.69(0.67–4.31)0.000 0.268 92.3	1.91(0.47–7.82)0.007 0.366 75.1
Source of control							
HB	9	1425/2048	1.39(0.93–2.08)0.000 0.106 90.8	1.52(1.03–2.24)0.000 0.037 84.7	1.84(0.61–5.56)0.000 0.280 87.0	1.54(0.97–2.44)0.000 0.064 89.6	1.52(0.63–3.66)0.000 0.352 79.8
PB	6	1623/1617	1.24(1.10–1.40)0.283 0.001 19.9	1.05(0.90–1.23)0.221 0.543 28.6	1.94(1.42–2.56)0.340 0.000 11.7	1.16(1.00–1.34)0.339 0.050 11.9	1.97(1.45–2.68)0.348 0.000 10.6

*M* Mutated allele; *W* Wide type allele*; HB* Hospital-based; *PB* Population-based; *P*_h_: value of *Q*-test for heterogeneity test; *P*: *Z*-test for the statistical significance of the OR

For the A/O combined SNP (AA/AO/OO) polymorphism, the O allele had a positive association with PTB risk in the total sample (heterozygote comparison: OR: 1.37, 95% CI: 1.06–1.7, *P* < 0.001 for heterogeneity, *P*: 0.018; dominant genetic model: OR: 1.41, 95% CI: 1.06–1.86, *P* < 0.001 for heterogeneity, *P*: 0.017; allelic contrast: OR: 1.33, 95% CI: 1.05–1.70, *P* < 0.001 for heterogeneity, *P*: 0.020, Fig. 3). In the subgroup analyses for different ethnicities, a similar significant association was detected for the Asian population (allelic contrast: OR: 1.26, 95% CI: 1.04–1.52, *P*: 0.001 for heterogeneity, *P*: 0.017, Fig. 3; heterozygote comparison: OR: 1.24, 95% CI: 1.01–1.52, *P*: 0.005 for heterogeneity, *P*: 0.044; dominant genetic model: OR: 1.28, 95% CI: 1.04–1.57, *P*: 0.003 for heterogeneity, *P*: 0.021, Fig. 3). Finally, in the subgroup analyses for different sources of control, PTB risk was significantly and positively associated with PB (e.g., allelic contrast: OR: 1.24, 95% CI: 1.10–1.40, *P*: 0.283 for heterogeneity, *P*: 0.001, Fig. 4) and HB studies (e.g., heterozygote comparison: OR: 1.52, 95% CI: 1.03–2.24, *P* < 0.001 for heterogeneity, *P*: 0.037, Fig. 5). In addition, no associations were observed for either rs7096206 or rs7095891, which indicated that heterogeneity might exist for these two SNPs (Table 2).

### Publication bias and sensitivity analysis

**Fig. 6:**
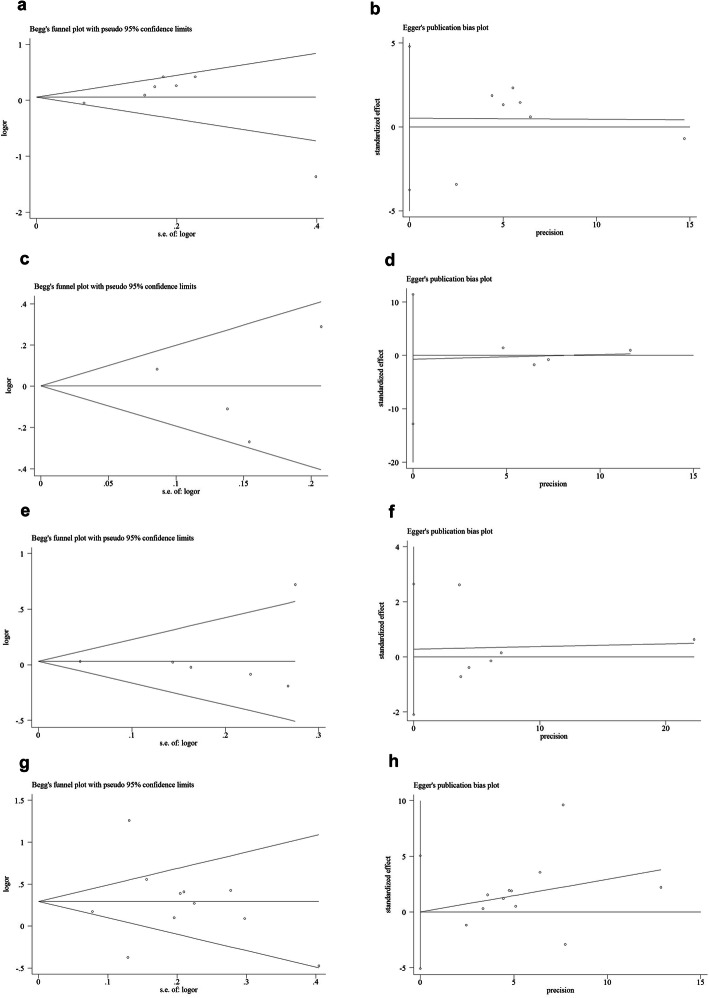
Begg’s funnel plot for publication bias test (allelic contrast: **a** of rs7096206 [*z* = − 0.19, *P* = 0.851]; **c** of rs11003125 [*z* = − 0.21, *P* = 0.835]; **e** of rs 7,095,891 [*z* = − 1.65, *P* = 0.099] and **g** of A/O combined SNP [*z* = − 0.74, *P* = 0.458]). Each point represents a separate study for the indicated association. Log [OR], natural logarithm of OR. Horizontal line, mean effect size. Egger’s publication bias plot (allelic contrast: **b** of rs7096206 [*t* = 0.56, *P* = 0.592]; **d** of rs11003125 [*t* = − 0.25, *P* = 0.812]; **f** of rs 7,095,891 [*t* = − 0.12, *P* = 0.912] and **h** of A/O combined SNP [*t* = − 0.02, *P* = 0.988])

**Fig. 7:**
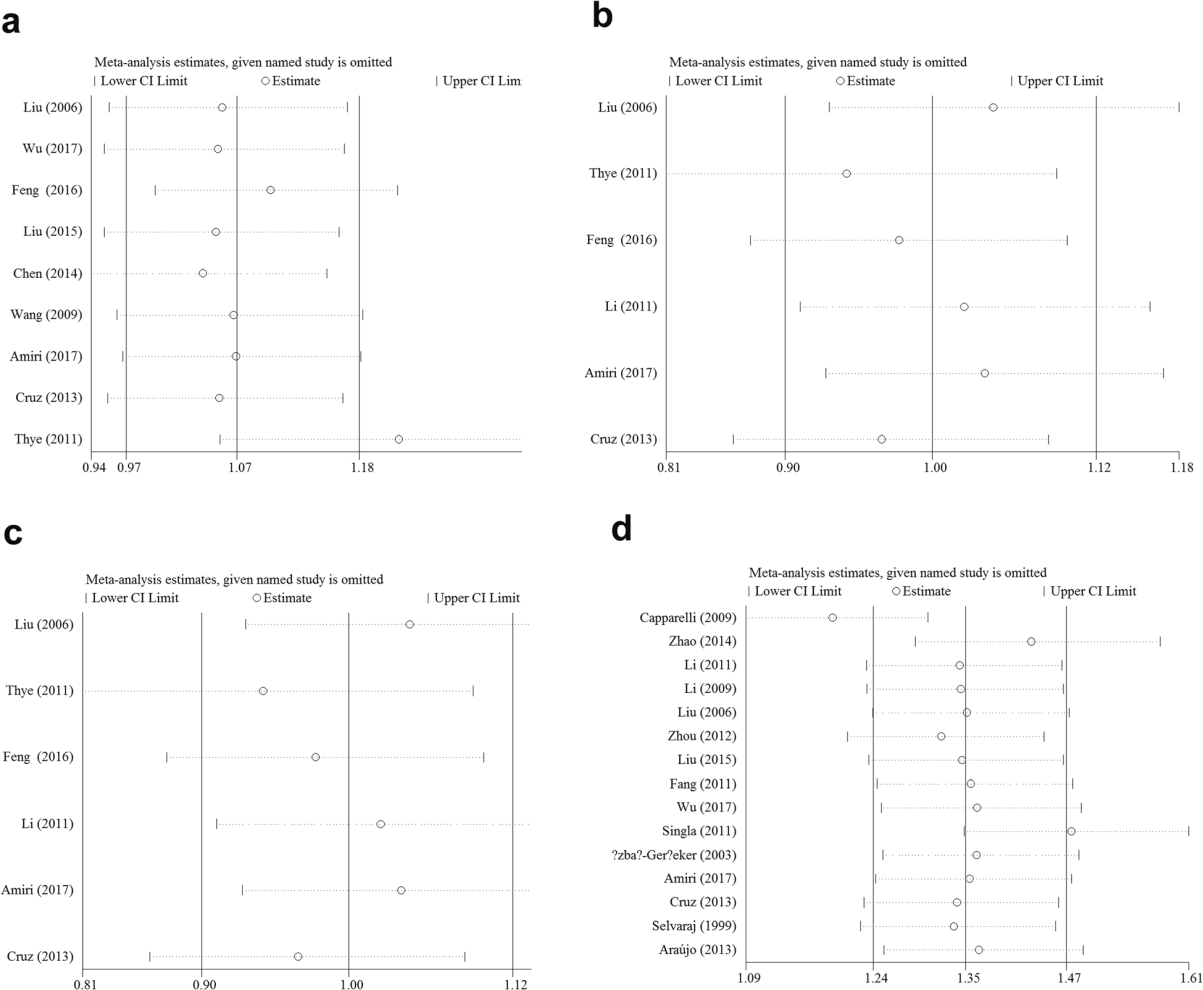
Sensitivity analysis between 4 SNPs in *MBL2* and PTB risk (allelic contrast: **a** for rs7096206; **b** for rs11003125; **c** for rs 7,095,891 and **d** for A/O combined SNP)

Begg’s test and Egger’s test were used to evaluate the publication bias of the included literature. The shape of the funnel plot did not show obvious asymmetry, and Egger’s test did not indicate publication bias (Fig. 6a-h, Table 3). We used sensitivity analysis to determine whether changes in a single study affected the outcome. For rs7096206 and O/A SNPs, two separate studies (Thye et al. for rs7096206, Fig. 7a and Capparelli et al. for O/A SNP, Fig. 7d) may have influenced the total OR according to the sensitivity analysis (data not shown).

**Table 3:** Publication bias tests (Begg’s funnel plot and Egger’s test for publication bias test) for 4 SNPs in *MBL2.*

Egger’s test						Begg’s test	
Genetic type	Coefficient	Standard error	t	***P*** value	95%CI of intercept	z	***P*** value
**rs7096206**							
X-allele vs. Y-allele	0.719	1.282	0.56	0.592	(−2.312,3.751)	0.1	0.917
XY vs. YY	0.597	1.084	0.55	0.599	(−1.967,3.161)	−0.1	1
XX vs. YY	0.149	0.429	0.35	0.739	(− 0.867,1.165)	0.31	0.754
XX + XY vs. YY	0.625	1.12	0.56	0.594	(−2.024,3.274)	−0.1	1
XX vs. XY + YY	0.147	0.434	0.34	0.744	(−0.878,1.172)	0.31	0.754
**rs11003125**							
L-allele vs. H-allele	−0.597	2.35	−0.25	0.812	(−7.122,5.928)	0	1
LH vs. HH	−0.477	0.808	−0.59	0.587	(−2.721,1.768)	0.38	0.707
LL vs. HH	1.899	1.558	1.22	0.29	(−2.426,6.226)	0.75	0.452
LL + LH vs. HH	−0.495	0.899	− 0.55	0.611	(−2.993,2.002)	0.75	0.452
LL vs. LH + HH	−0.15	2.385	−0.06	0.953	(−6.772,6.472)	0	1
**rs7095891**							
Q-allele vs. P-allele	−0.09	0.781	−0.12	0.912	(−2.099,1.917)	1.5	0.133
QP vs. PP	−0.068	0.763	−0.09	0.932	(−2.031,1.893)	1.2	0.23
QQ vs. PP	−0.064	0.15	−0.43	0.687	(−0.451,0.322)	0.3	0.764
QQ + QP vs. PP	−0.077	0.775	−0.1	0.924	(−2.069,1.914)	1.2	0.23
QQ vs. QP + PP	−0.065	0.149	−0.44	0.68	(−0.448,0.317)	0.3	0.764
**AA/AO/OO**							
A-allele vs. O-allele	−0.026	1.703	−0.02	0.988	(−3.706,3.653)	0.69	0.488
AO vs. OO	0.469	1.407	0.33	0.744	(−2.571,3.511)	0.4	0.692
AA vs. OO	0.113	0.422	0.27	0.792	(−0.798,1.025)	1.39	0.166
AA+AO vs. OO	0.513	1.398	0.37	0.72	(−2.507,3.533)	0.59	0.533
AA vs. AO + OO	0.091	0.436	0.21	0.839	(−0.852,1.033)	1.39	0.166

## Discussion

Previous studies on the incidence of TB primarily focused on tubercle bacilli and the effects of environmental risk factors (such as sex, previous group TB, smoking status, drinking status, dominant status, age, group size, rainfall, immigration, number of eligible rovers, public health, economic, conservation importance). In recent decades, the effect of host susceptibility genes on TB has been increasingly recognized along with the development of genetic susceptibility. However, recent studies on the associations between SNPs in *MBL2* and TB have produced different and even contradictory results. Some studies have indicated that mutations in the promoter and exon 1 of *MBL2* may lead to the decline of MBL expression in the serum, while lower serum MBL levels can increase infections caused by tubercle bacilli [40, 54], indicating that polymorphisms in *MBL2* may exert a protective effect against TB. Other studies have indicated that higher serum levels of MBL can reduce tubercle bacilli infections, which are associated with wild-type *MBL2* alleles [5, 27, 55]. These studies suggest that *MBL2* variants may increase the risk for TB.

Several meta-analyses have focused on the relationships between *MBL2* polymorphisms and susceptibility to TB; however, each meta-analysis has its own conclusion and merits. Cao et al. analyzed 22 studies to assess the effect of *MBL2* polymorphisms on TB risk. The rs1800451 polymorphism was associated with decreased TB risk in both the total sample and in some ethnic groups; in addition, A/O, rs7096206 and rs1800450 were likely only related to risk in some ethnic groups [56]. The analysis did not differentiate between the total sample and PTB subgroups. Tong et al. suggested that rs1800450 and rs5030737 polymorphisms were risk factors for susceptibility to TB; nevertheless, rs7095891 and rs1800451 polymorphisms acted as protective factors against TB [57]. Their study did not analyze the differences between the total sample and subgroups of TB. Denholm et al. [16] examined 12 case-control studies of HIV-negative patients and two studies of HIV-positive patients to determine the association of the *MBL2* structural gene variants (B, C and D, referred to collectively as O, and A is the wild-type) with TB susceptibility. They did not find a significant association between the *MBL2* genotype and PTB infection. By contrast, a meta-analysis of four studies examining MBL levels and susceptibility to TB found a significant association of high MBL levels with susceptibility to TB, although increased serum MBL levels due to the acute-phase reaction could not be ruled out. In addition, Areeshi et al. [58] found a statistically significant association of the C (rs1800451) alleles and genotypes with a reduced risk of TB in the overall population. No significant associations were observed in other variant sites (such as rs1800450, rs5030737, rs7096206, rs11003125, rs7095891 and combined rs1800450 O-alleles). Stratified analysis by ethnicity showed a decreased risk of TB in the African population for rs1800450 (B) and rs1800451 (C) alleles and genotypes. However, no association was observed between other *MBL2* polymorphisms and TB risk in Asians. The results indicated a protective role of alleles B and C in TB infection. Finally, Shi et al. [59] indicated that individuals carrying the *MBL2* codon 54 B allele had an increased risk of TB compared with AA homozygotes, whereas rs7095891 was possibly not associated with TB risk in Chinese.

To our knowledge, the current study is an updated systematic analysis exploring the relationships between *MBL2* variants and PTB susceptibility. This analysis involved approximately 12,052 patients with PTB and 13,905 healthy samples. The most important finding of our study was that the rs11003125 L-allele and the A/O combined SNP were risk factors for PTB susceptibility in the HB subgroup, which was similar to findings from a previous meta-analysis. The O allele was also a risk factor for PTB in the Asian and PB subgroups. The aforementioned conclusions were novel concepts that have not been found in previously published meta-analyses.

The above contradictory results from previous meta-analyses further emphasize the controversy about the effect of *MBL2* variants on susceptibility to TB. One possible explanation for this effect is that different polymorphisms may have different effects on gene function, resulting in changes in PTB susceptibility. Second, the complex interaction between several genetic and environmental factors may involve the development of PTB. We think these conflicting results among studies and different populations suggests linkage disequilibrium with other nearby genes (e.g., surfactant proteins A1, A2 and D [60] previously associated with TB) rather than a causative association between *MBL2* variants and PTB. Third, it is now widely accepted that differences in ethnicities between cases and control measures may be a source of confusion in the compilation of studies. Fourth, research with “negative” results takes longer to publish due to the time-lag bias, and positive research results are published much faster. Fifth, small studies of with “negative” results have never been published, and small studies of similar quality with “positive” results will also be shown in the literature [61–63]. Sixth, rs7096206, rs11003125 and rs7095891 SNPs were not analyzed in the previous three meta-analyses; our study was the first to analyze these SNPs. Furthermore, we focused on PTB but not on total TB or extrapulmonary TB (EPTB), in contrast to previous meta-analyses.

Some limitations in our study should be noted. Initially, we collected all eligible studies; however, the sample size of these studies is not yet large enough, especially in certain ethnic groups. Therefore, not only is the likelihood of I/II type errors high, but there is insufficient statistical capacity to assess the correlations between the 7 SNPs and PTB risk. Second, serum MBL concentration was not assessed in our study, which would have been helpful for detecting and understanding the mechanism of SNPs in the *MBL2* gene. Third, other factors such as age, sex, smoking, familial history, disease stage, specific environmental factors and lifestyles should be included. Fourth, only one article [19] included the subgroups of HIV- and HIV+, anti-TNF drugs, and DM; these groups were not evaluated in other included studies, so we could not analyze the associations within the above groups because of missing information. Fifth, the included studies had a high amount of heterogeneity. In addition, we cannot know whether patients had latent tuberculosis. Finally, all included studies were epidemiological surveys; there were no plausible biological hypotheses or mechanistic studies. We aimed to determine whether there is a relationship between *MBL2* structural gene variants and susceptibility to PTB. Further studies should aim to overcome these limitations.

In summary, our study indicated that the rs11003125 and A/O-combined SNPs in *MBL2* may be related to PTB risk. Larger sample sizes and additional gene-environment interactions should be considered in future studies.

## Data Availability

All the data generated in the present research is contained in this manuscript.

## Abbreviations

MBL: Mannose-binding lectin
PTB: Pulmonary tuberculosis
ORs: Odds ratios
CIs: Confidence intervals
SNPs: Single nucleotide polymorphisms
HWE: Hardy-Weinberg equilibrium
